# Associative learning shapes visual discrimination in a web-based classical conditioning task

**DOI:** 10.1038/s41598-021-95200-6

**Published:** 2021-08-03

**Authors:** Yannik Stegmann, Marta Andreatta, Paul Pauli, Matthias J. Wieser

**Affiliations:** 1 Department of Psychology (Biological Psychology, Clinical Psychology, and Psychotherapy), University of Würzburg, Marcusstraße 9-11, 97070 Würzburg, Germany; 2 Department of Psychology, Education, and Child Studies, Erasmus University Rotterdam, Rotterdam, The Netherlands; 3 Center for Mental Health, Medical Faculty, University of Würzburg, Würzburg, Germany

**Keywords:** Psychology, Sensory processing, Classical conditioning, Fear conditioning, Visual system

## Abstract

Threat detection plays a vital role in adapting behavior to changing environments. A fundamental function to improve threat detection is learning to differentiate between stimuli predicting danger and safety. Accordingly, aversive learning should lead to enhanced sensory discrimination of danger and safety cues. However, studies investigating the psychophysics of visual and auditory perception after aversive learning show divergent findings, and both enhanced and impaired discrimination after aversive learning have been reported. Therefore, the aim of this web-based study is to examine the impact of aversive learning on a continuous measure of visual discrimination. To this end, 205 participants underwent a differential fear conditioning paradigm before and after completing a visual discrimination task using differently oriented grating stimuli. Participants saw either unpleasant or neutral pictures as unconditioned stimuli (US). Results demonstrated sharpened visual discrimination for the US-associated stimulus (CS+), but not for the unpaired conditioned stimuli (CS−). Importantly, this finding was irrespective of the US’s valence. These findings suggest that associative learning results in increased stimulus salience, which facilitates perceptual discrimination in order to prioritize attentional deployment.

## Introduction

Learning to detect threat-predicting stimuli is a fundamental function for supporting adaptive behavior in ever-changing environments. Recent theories propose that threat learning prompts changes in sensory processing to improve discrimination of threat- and safety-related signals. These changes can manifest on every stage of the sensory system hierarchy and have already been documented for a wide range of modalities. For example, Kass et al.^1^ observed facilitated synaptic output of olfactory sensory neurons in rodents after selectively pairing odors with electrical stimuli. Similar results could be demonstrated in humans by using initially indistinguishable odor cues in an aversive learning paradigm. Functional imaging analyses showed that olfactory threat learning modified neural organization in the primary olfactory cortex, which was accompanied by enhanced behavioral discrimination of threat-related odors^2^. Following threat learning, auditory cortex neurons showed enhanced responsiveness to threat-predicting tones in rats^3^, rabbits^4^, and cats^5^. These findings are paralleled by results of studies measuring neural activity of the human auditory cortex during threat learning by the means of functional magnetic resonance imaging (fMRI)^6^, magnetoencephalography (MEG)^7^, and electroencephalography (EEG)^8^, demonstrating unequivocal evidence for increased response amplitudes to threat-predicting stimuli.

Studies investigating the visual sensory system yielded comparable results^9^. Using EEG as a direct measure of visuocortical activity, numerous studies demonstrated converging evidence for enhanced sensory processing of threat-related features of visual stimuli. Mirroring the organization of visual receptive fields, threat-related sensory amplification has been reported for visual stimuli differing in orientation^10,11^, spatial frequency^12^, location^13^, and color^14^. However, enhanced visuocortical responses have also been observed for more complex visual stimuli, like geometric symbols^15,16^, pictures of virtual rooms^17^ and facial identities^18,19^.

Together, these studies support the notion that sensory systems are dynamically adapting in response to environmental cues signaling motivational significance^20^. However, the extent to which these adaptions in the sensory cortices are accompanied by changes in behavioral performance remains elusive, and recent studies investigating the psychophysics of visual and auditive discrimination during aversive learning showed mixed results. Contrary to expectations, Resnik, et al.^21^ found an increase in discrimination thresholds using auditive stimuli paired with aversive odors or sounds. The authors suggest that aversive learning prompts generalization processes in sensory systems to widen responses to upcoming danger, thus lowering visual discrimination. In a similar line of studies, the authors observed the same pattern for visual stimuli differing in orientation or contrast followed by aversive picture stimuli^22^. In contrast, Rhodes, et al.^23^ showed decreased discrimination thresholds using differently oriented grating stimuli followed by aversive noise blasts. Importantly, this improved visual discrimination was specific for the grating stimulus paired with the aversive sound and was not found for other orientations.

Despite their methodological similarities these studies show divergent results. Both lines of studies employed a differential fear conditioning paradigm and measured the change of discrimination acuity using a psychophysical staircase approach to identify the just noticeable difference threshold (JND). Even though the JND is a well-established psychophysical index^24^, it may have a limited use in capturing sensory changes in aversive learning paradigms. First, the JND is only a point measure of discrimination acuity, thus obscuring potential nongradual effects on visual discrimination along the perceptual continuum. These nongradual effects have already been identified, for example, for visuocortical activation patterns during aversive generalization learning paradigms using grating stimuli^10,25^ or faces^18^. In this line of studies, visuocortical activity as an index of sensory engagement increased with increasing similarity to the threat-related stimulus for all except for the most similar generalization stimuli, which elicited decreased activity. Thus, the effect of aversive learning on visuocortical responding did not follow the expected increasing gradient for the feature similarity continuum. In these studies, this nongradual effect is discussed in terms of lateral inhibition among feature sensitive neurons in the primary visual area, which has been suggested to be associated with increased sensory perception for the stimuli most similar to the threat-related stimulus^10^. However, point measures like the JND do not allow for a quantification of these differences in sensory perception among the feature similarity continuum. Second, the JND procedure varies in the number of unreinforced presentations of the conditioned stimulus, depending on the participant’s response pattern. This might be problematic as longer procedures are more affected by the temporal dynamics of extinction learning. This is aggravated by the observation that threat-related visuocortical sharpening extinguishes rapidly^10^.

To overcome these issues, we used a yes–no task to measure visual discrimination along a continuum of radial distance in steps of 1° before and after differential aversive learning. The goals of this study were threefold: (1) Based on recent studies delivering fear conditioning tasks remotely^26,27^, we aimed at establishing a web-based discrimination and aversive learning task, exploiting the benefits of quickly and economically collecting data from large samples outside of the laboratory context. (2) Given the mixed results regarding how aversive learning influences discrimination acuity, we tested the hypothesis that aversive learning changes visual discrimination for the threat-related stimulus (CS+). In particular, we expected that only participants who were conditioned with an aversive US, would demonstrate either improved^23^ or decreased^21^ discrimination acuities for CS+, but not for CS− orientations. In contrast, participants, who were confronted with the same discrimination task, but saw neutral US during associative learning trials, should show no changes in visual discrimination. (3) To further test how discriminative learning impacts discrimination acuity, we included two further groups that did the same discrimination task as the differential learning groups but saw six additional generalization stimuli (GS) in 10° steps around the CS+ (− 30°, − 20°, − 10°, 10°, 20°, 30°; )^10^ during associative learning with either aversive or neutral US. We expected that the group confronted with aversive US exhibit even higher discrimination acuities after learning, since they experience that only the CS+ is followed by an US, while all other GS, even those perceptually similar to the CS+ , signal safety and thus are required to pay closer attention to differences in orientation. Again, we expected changes in visual discrimination to occur only for aversive US-associated, but not for neutral US-associated orientations.

## Methods

### Preregistration and online data

The hypotheses and methods of this study were preregistered. The preregistration, all data and analyses are publicly available at https://osf.io/4cz2e.

## Participants

In total, 336 individuals started the study of whom 205 (65.2%) participants (169 females) with a mean age of 23.8 ± 7.1 years completed the web-based paradigm. Data from the 131 dropouts were not saved due to the settings of the website and thus could not be analyzed. Participants were required to be older than 18 years and be free of a lifetime history of mental disorders (as assessed by self-report). After completing demographic and anxiety questionnaires, participants were randomly allocated to one of six experimental groups. There were four groups that completed a standard differential fear conditioning paradigm (Diff), while two groups underwent a modified fear generalization paradigm (Gen). Two of the differential learning groups were tested on the unpaired conditioned stimulus (control group, c) in the discrimination task. Groups were either presented with aversive (+) or neutral (−) US. Characteristics for the six resulting groups are summarized in Table 1. All experimental procedures were approved by the ethics committee of the Department of Psychology at the University of Würzburg. Procedures were in agreement with the Declaration of Helsinki (Version 2008). All participants provided informed consent online. They received either course credits or could join a lottery for one out of five 15€ coupons as compensation.

**Table 1:** Summary of group characteristics.

	Groups						F(5,199)	*p*
	Diff+	Diff−	Gen+	Gen−	cDiff+	cDiff−		
n	31	37	37	36	32	32		
Women (%)	77.4	94.6	70.3	86	81.2	84.4	1.73	0.129
Age	23.7 (6.1)	26.0 (10.3)	25.4 (8.7)	23.9 (4.7)	22.4 (5.8)	20.6 (2.7)	2.71	0.021
BAI	11.3 (10.1)	12.3 (9.4)	13.2 (9.9)	11.5 (11.0)	15.4 (13.3)	13.5 (10.5)	0.64	0.669
IUS	48.1 (14.6)	46.5 (12.6)	49.1 (15.8)	45.8 (15.6)	49.8 (13.1)	48.8 (11.1)	0.43	0.827
US arousal	70.1 (16.0)	26.5 (20.7)	70.4 (18.5)	23.3 (17.9)	69.7 (24.4)	28.5 (21.0)	50.5	< 0.001
US unpleasantness	71.4 (21.1)	49.4 (8.8)	75.7 (15.1)	47.9 (9.3)	74.4 (15.2)	44.4 (14.9)	35.6	< 0.001
Memory scores	8.5 (3.3)	9.7 (1.5)	8.5 (3.0)	9.1 (2.0)	9.25 (1.9)	8.56 (3.1)	1.41	0.224

Diff = differential learning group, Gen = generalization learning group, c = control discrimination task (CS −), +  = aversive US, −  = neutral US, BAI = Becks Anxiety Inventory, IUS = Intolerance of Uncertainty Scale. Numbers indicate means (± S.D.).

## Materials

Circular black-and-white sinusoidal grating stimuli (10 Hz spatial frequency) filtered with a Gaussian-envelope (i.e., Gabor-patch) with maximum contrast of 100% at center were used as conditioned stimuli for the conditioning procedure and as target stimuli for the visual discrimination task. Aversive and neutral picture stimuli served as unconditioned stimuli (US). All pictures were extracted from the OASIS data set^28^. Ten high arousing/unpleasant (I26, I209, I208, I714, I276, I855, I210, I496, I287, I440) and ten low arousing/neutral (I182, I597, I602, I673, I181, I891, I596, I195, I632, I594) pictures were chosen according to their normative ratings for arousal and valence. To ensure that participants perceived the aversive or neutral picture stimuli as such, they were asked to rate the picture stimuli for subjective arousal (“As how arousing do you perceive this picture?”, 0 = low arousing, calm to 100 = very arousing) and valence (“As how pleasant/unpleasant do you perceive this picture?”, 0 = very pleasant, 100 = very unpleasant) using visual analogue scales at the end of the experimental session. All stimuli were presented centrally on a grey background (RGB = 128, 128, 128). Please note, that we could not control the participants’ monitor setup in this web-based study. However, the experiment did not run on tablets or mobile devices.

### Design

**Figure 1:**
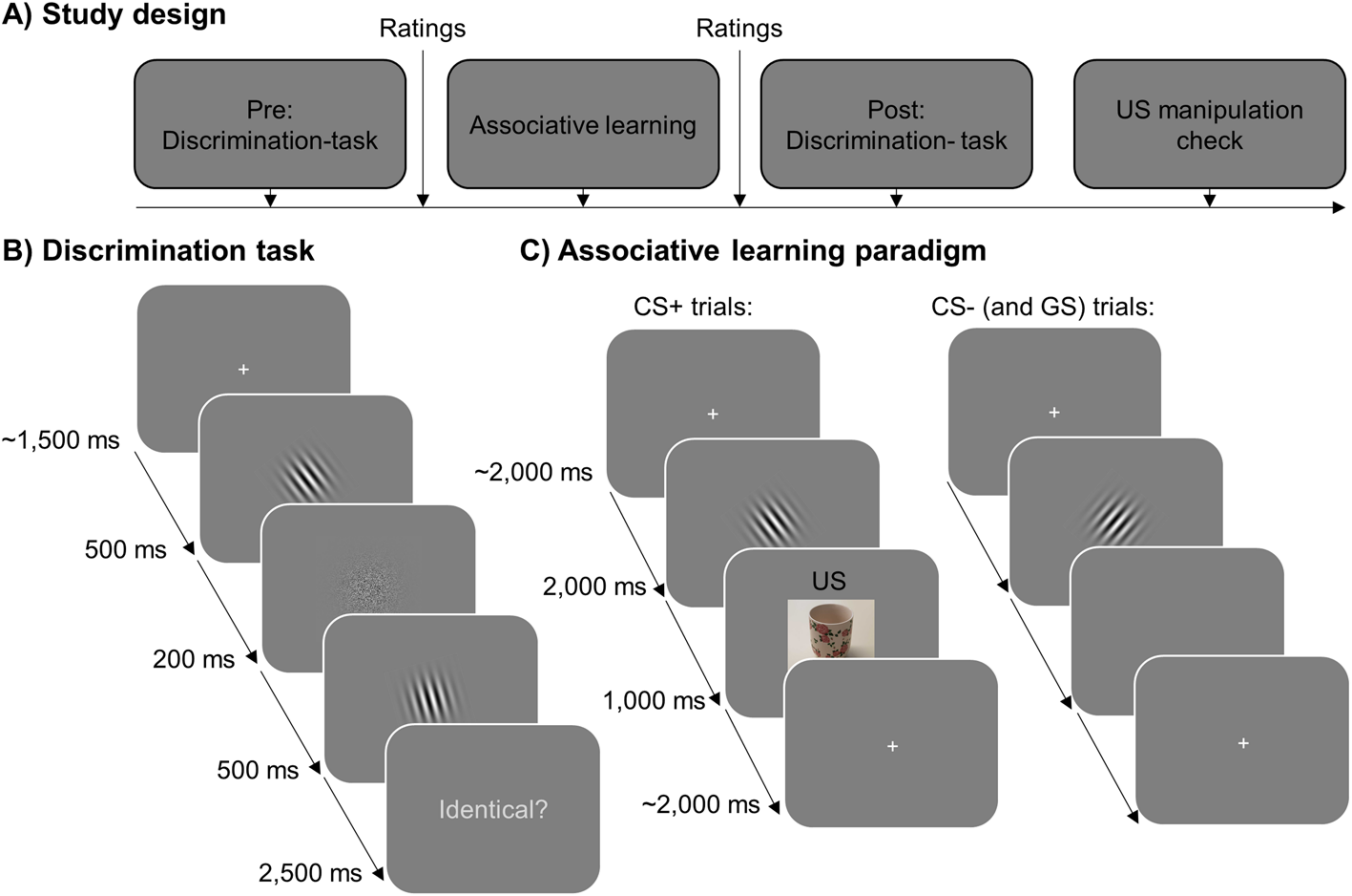
(**A**) Flowchart of the general experimental design. (**B**) Trial structure and timing of the visual discrimination task. Crucially, at least one of the grating stimuli was always the later CS+ (CS− for control groups, cDiff+ and cDiff −). (**C**) Trial structure and timing of the associative learning task. CS+ trials were always followed by a 1.000 ms presentation of a US (either a neutral or an unpleasant picture stimulus), while CS− trials (and all GS trials for generalization groups, Gen+ and Gen−) remained unpaired. Note: The US depicted in this figure is an example and differs from the pictures used in the original experiment.

**Figure 2:**
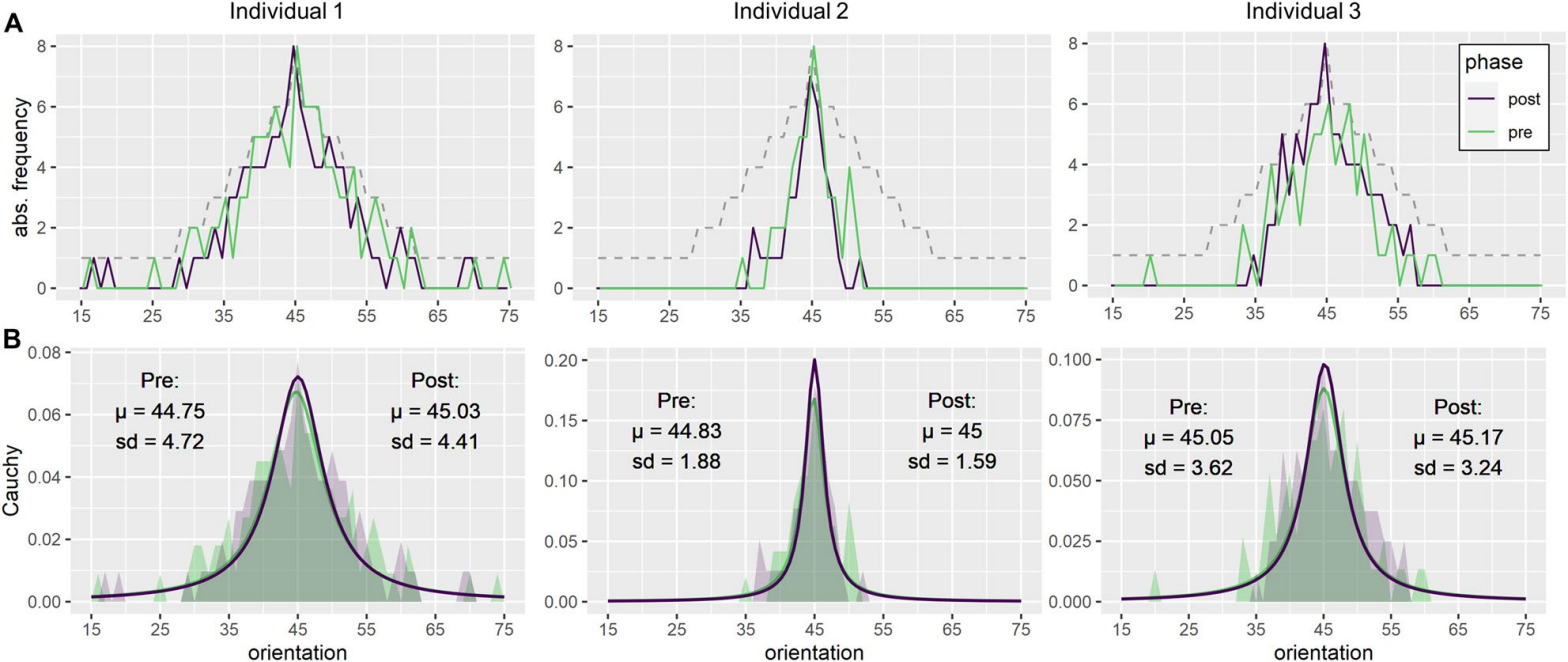
(**A**) Three examples of individual response data from the visual discrimination task. The green and purple lines represent the sum of ‘identical’-responses per condition during the pre- and post-task, respectively. The dotted line depicts the number of total presentations per condition. (**B**) Bold lines show the fitted Cauchy-distributions over the behavioral response data (shaded areas) prior and after associative learning. The extracted means (µ) and standard deviations (SD) are noted in the diagram.

After giving informed consent, participants completed German versions of a demographic questionnaire, Becks-Anxiety-Inventory (BAI)^29^ and Intolerance of Uncertainty scale (UI-18)^30,31^, using an online survey platform (www.soscisurvey.de). They were then redirected to www.pavlovia.org, where the main experiment took place (see Fig. 1A). The discrimination task consisted of 160 trials, each starting with a 1500–2500 ms presentation of a centrally presented fixation cross, before the first Gabor-patch was presented for 500 ms (see Fig. 1B). The Gabor-patch was masked by a scrambled stimulus (200 ms) before the second Gabor-patch was presented for 500 ms. One of the Gabor-patches was always the target stimulus (CS+ in the experimental groups, CS− in the control groups). The target was presented either as first or second Gabor-patch in 50% of the trials, respectively. Importantly, there were eight trials, in which both Gabor-patches were presented in the target orientation. Beginning with the onset of the second Gabor-patch, participants had up to 3000 ms for responding weather the Gabor-patches were identical (Press ‘X’) or not (Press ‘N’). The orientation of the Gabor-patches varied between − 30° and 30° in steps of 1° around the target stimulus. Since we assumed that effects of aversive learning on visual discrimination would be most likely evident in orientations perceptually similar to the target orientation, the number of presentations per orientation followed a normal distribution around the target orientation (i.e., more presentations of orientations close to the target orientation). Crucially, the number of presentations per orientation was determined a priori and was the same for all participants. The absolute frequency of the final distribution is illustrated as dashed lines in Fig. 2A. Based on this distribution, the presentation order was pseudo-randomly shuffled for each participant and timepoint. Participants did not receive feedback except for 20 practice trials at the beginning of the experiment.

Next, all participants completed an aversive learning paradigm according to their group condition. There were four groups that completed a standard differential fear conditioning paradigm (Diff+ , Diff− , cDiff+ , cDiff −), while two additional groups underwent a modified fear generalization paradigm (Gen+ , Gen-). Participants were either presented with aversive (+) or neutral (−) USs. The standard differential learning paradigm consisted of 40 CS+ presentations and 40 CS− presentations (80 trials in total). Crucially, the differential learning groups that were psychophysically tested on the CS− (cDiff+, cDiff−) completed the same learning task as the groups that were tested on the CS+ (Diff+ , Diff−). In contrast, the generalization learning groups (Gen+ , Gen−) were presented with 10 trials of each CS+ , CS− and six generalization stimuli (GS) in 10° steps around the CS+ (− 30°, − 20°, − 10°, 10°, 20°, 30°)^10^, also resulting in 80 total trials. For all groups, every CS+ presentation was followed by a presentation of an US (100% reinforcement rate), while the CS − (and all GS in generalization groups) remained unpaired. Importantly, five of the ten aversive picture stimuli served as US for the aversive learning groups (Diff+ , Gen+ , cDiff+), whereas five of the ten neutral picture stimuli were used as US for the neutral learning groups (Diff− , Gen− , cDiff −). The remaining five pictures of the same category were used in the US memory task at the end of the experiment. The choice of US was counter-balanced between participants. During each trial, the CS was presented for 2000 ms and was followed by a 1000 ms presentation of the US in case of the CS+. The duration of the ITI varied between 2000 and 3000 ms (see Fig. 1C). At the beginning of the acquisition phase, all participants were told to watch the stimuli carefully and that neutral or unpleasant pictures (depending on the group) will be presented in addition to the grating-stimuli, but they did not receive explicit instructions about the CS-US contingencies.

Before and after associative learning, every participant rated the CS and all GS for subjective threat (‘How threatening do you perceive this stimulus?’) on a visual analogue scale ranging from 0 = ‘not threatening at all’ to 100 = ‘very threatening’. US-expectancy ratings (‘How much do you expect the presentation of a picture after this stimulus?’; 0 = ‘not at all’; 100 = ‘very likely’) were collected after associative learning only.

Afterwards, participants repeated the visual discrimination task. At the end of the experiment, participants were submitted to an US-validation task, consisting of the US valence- and arousal-rating and a memory task. During the memory task, participants were presented the five picture stimuli which had served as US previously, in addition to the five novel stimuli. For each stimulus, participants had to answer (yes/no) if they had encountered this picture previously during the experimental session. Each correct answer was counted and summarized, resulting in a memory score of zero to ten correctly remembered stimuli.

### Discrimination task and data analysis

For behavioral analysis, all ‘identical’-responses were collected and summarized per condition, phase and individuum (see Fig. 2A). To obtain a single measure of discrimination acuity for each phase and individuum, unimodal distributions were fitted to the individual response data (see Fig. 2B). Then, individual means and standard deviations of the Cauchy-distributions were extracted, using the ‘fitdist’-algorithm of the ‘fitdistrplus’-package^32^ in R (version 4.0.2)^33^. Cauchy-distributions were used instead of standard-normal distributions, as they are more robust to outliers for conditions at the ends of the continuum, which were presented only once^34^. Importantly, the width of a Cauchy-distribution is represented by its standard deviation. Thus, smaller standard deviations indicate better visual discrimination. Therefore, the standard deviations of these distributions serve as an index of discrimination ability and the change of standard deviations can be used to measure the impact of associative learning on discrimination acuity. Extracted standard deviations were log-transformed to account for skewedness in the distribution of the data. Shapiro–Wilk tests of normality still indicated left-skewed distributions for the pre-task, *w*(205) = 0.981, *p* = 0.009. However, visual inspection of the data and analysis of the post-task, *w*(205) = 0.991, *p* = 0.23, and most importantly, of the difference between pre- and post-task, *w*(205) = 0.988, *p* = 0.09, demonstrated satisfactory normality of the distributions of the log-transformed standard deviations. Thus, log-transformed standard deviations were used for all statistical analyses.

### Statistical analysis

Statistical analyses and figure processing were conducted with the freely available open-source software R 4.0.2^33^.

Aggregated data from the discrimination task were submitted to two separate approaches of analyses**.** (1) To compare the different learning paradigms, we conducted a 2 (learning type: differential vs. generalization learning) × 2 (aversiveness: neutral vs. aversive US) × 2 (phase: pre vs. post) ANOVA on the individual standard deviations. For this analysis, we included data from the Diff+ , Diff− , Gen+ and Gen− groups. (2) To investigate the effect of US-contingency on visual discrimination, we run a 2 (target: CS+ vs CS −) × 2 (aversiveness: neutral vs. aversive) × 2 (phase: pre vs. post) ANOVA. Here, we included data from the Diff+ , Diff− , cDiff+ and cDiff− groups.

Similarly, for analyses of US-expectancy and threat ratings, we conducted (1) 2 (learning type: differential vs. generalization learning) × 2 (aversiveness: neutral vs. aversive US) × 8 (orientation: − 30°, − 20°, − 10°, CS+ , + 10°, + 20°, + 30°, CS−) ANOVAs and (2) 2 (target: CS+ vs CS −) × 2 (aversiveness: neutral vs. aversive US) × 8 (orientation: − 30°, − 20°, − 10°, CS+ , + 10°, + 20°, + 30°, CS−) ANOVAs. Please note that for rating analyses, the factor phase was omitted since US-expectancy ratings were only collected after associative learning.

Significant effects were followed up with *t* tests. For rating analyses, post-hoc tests for the factor orientation were referenced against the CS− as is frequently done in the fear conditioning literature^35,36^. A significance level of 0.05 was used for all analyses and Greenhouse–Geisser correction was applied where appropriate^37^. Throughout this manuscript, corrected degrees of freedom, corrected *p* values and the partial η^2^ (*η*_*p*_^2^) or Cohen’s d (*d*) and their 95% confidence interval are reported^38^.

## Results

### Discrimination task

**Figure 3:**
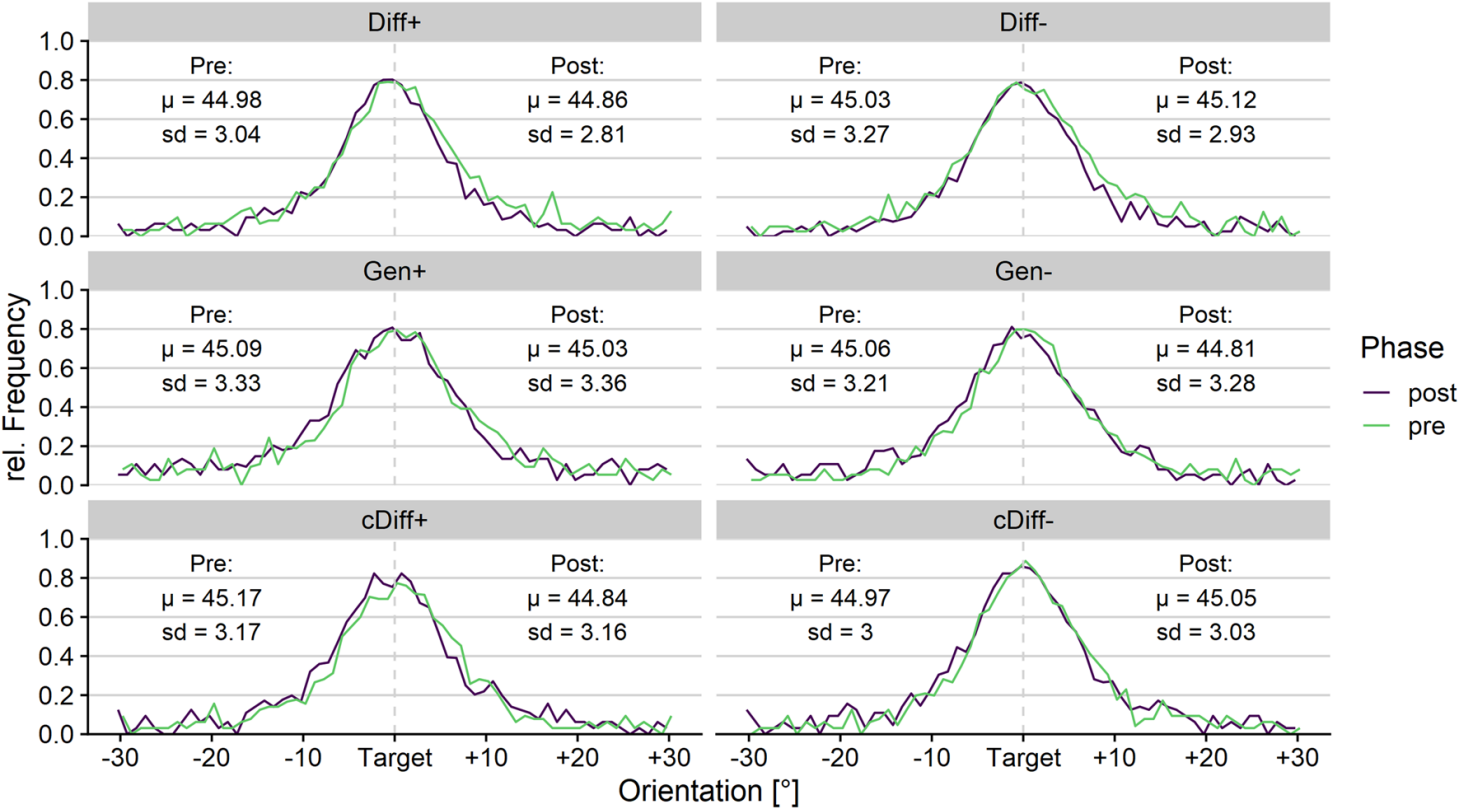
Group-averaged response distributions during the discrimination tasks. The proportion of ‘identical’-responses per orientation were calculated as relative frequency. The dotted vertical line represents the target orientation (CS+ for Diff and Gen, CS− for cDiff). The grand averages of the extracted means (µ) and standard deviations (SD) are noted in the diagram.

**Figure 4:**
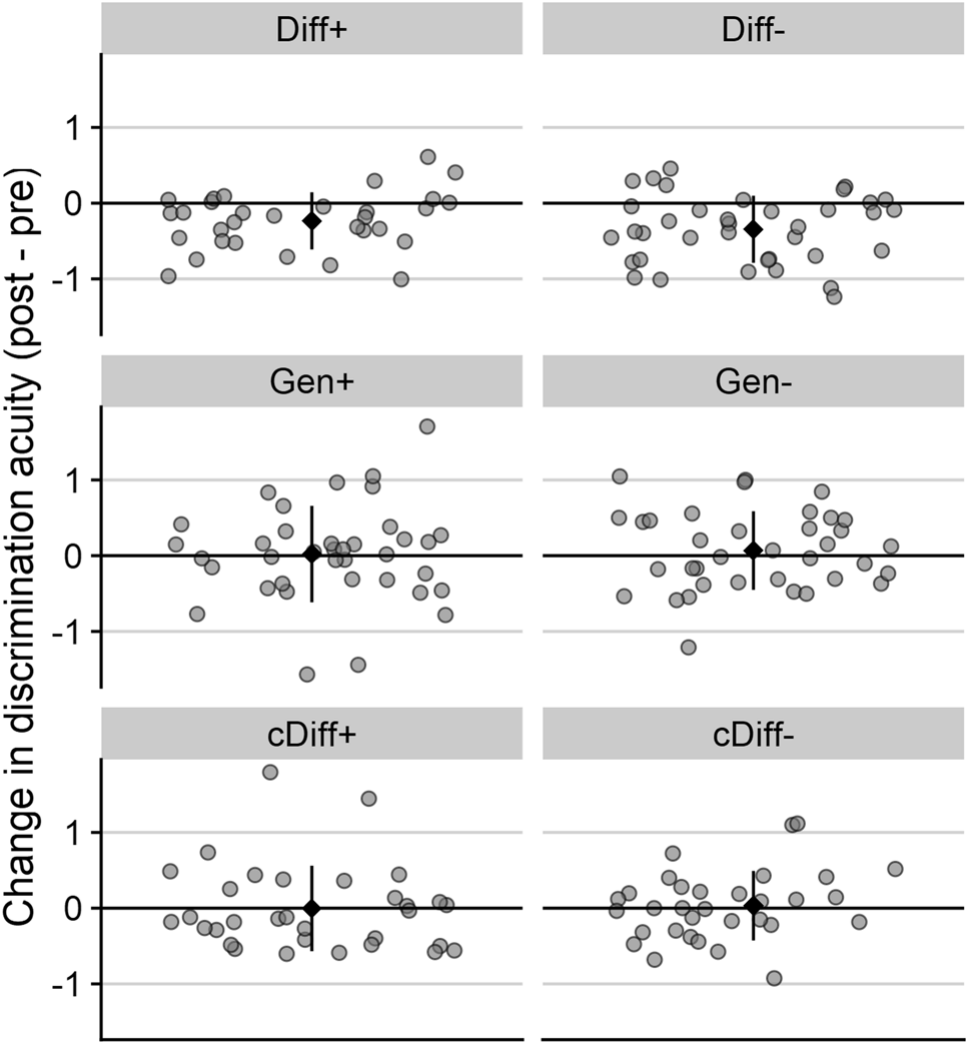
Individual change scores in discrimination acuity (post–pre standard deviation) per group. Positive/negative values indicate impaired/improved discrimination acuity, respectively. Black diamonds depict mean scores, while error bars represent aggregated standard deviations.

The 2 (learning type: differential vs. generalization learning) × 2 (aversiveness: neutral vs aversive US) × 2 (phase: pre vs. post) ANOVA of the standard deviations extracted from the discrimination task yielded a main effect of phase, *F*(1, 137) = 9.94, *p* = 0.002, *η*_*p*_^2^ = 0.07 [CI 0.01; 0.16], and a marginal main effect of learning type, *F*(1, 137) = 2.98, *p* = 0.086, *η*_*p*_^2^ = 0.02 [CI 0.00; 0.09], which were further qualified by an interaction effect between phase and learning type, *F*(1, 137) = 20.06, *p* < 0.001, *η*_*p*_^2^ = 0.13 [CI 0.04; 0.23]. Other effects were not significant, *p*s > 0.289. Post-hoc *t* tests revealed a decrease in standard deviations, and therefore, improved discrimination acuity after associative learning for the groups Diff+ , *t*(30) =  − 3.34, *p* = 0.002, *d* =  − 0.60 [CI − 0.98; − 0.21], and Diff − , *t*(36) =  − 4.78, *p* < 0.001, *d* =  − 0.79 [CI − 1.15; − 0.41], but not for Gen+ , *t*(36) = 0.53, *p* = 0.601, *d* = 0.09 [CI − 0.24; 0.41], and Gen − , *t*(35) = 0.76, *p* = 0.452, *d* = 0.13 [CI − 0.20; 0.45]. These results indicate sharpened visual discrimination after associative learning in the differential learning groups, but not in the generalization learning groups, independent of the US valence (see Figs. 3, 4).

To test the specificity of this effect, the differential learning groups were compared to the control groups, who underwent the same learning paradigm but were psychophysically tested on the CS− . The 2 (target: CS+ vs CS-) × 2 (aversiveness: neutral vs. aversive US) × 2 (phase: pre vs. post) ANOVA revealed a main effect of phase, *F*(1, 128) = 13.53, *p* < 0.001, *η*_*p*_^2^ = 0.10 [CI 0.02; 0.20], which was again further qualified by a significant interaction between target and phase, *F*(1, 128) = 12.76, *p* < 0.001, *η*_*p*_^2^ = 0.09 [CI 0.02; 0.19]. No other effect was significant, *p*s > 0.158. The control groups showed no changes in discrimination acuity due to associative learning, cDiff + : *t*(31) =  − 0.63, *p* = 0.535, *d* = − 0.11 [CI − 0.46; 0.24], cDiff− : *t*(31) = 0.63, *p* = 0.536, *d* = 0.11 [CI − 0.24; 0.46].

To exclude that changes in standard deviations are an effect of a general response bias, i.e., responding less to all orientations (including target orientations), we specifically compared the absolute number of ‘identical’-responses to the target-target trials before and after associative learning. Crucially, no group showed significant changes in the number of ‘identical’-responses to identical trials as a result of associative learning, Diff+ : *t*(30) = 0.37, *p* = 0.712; Diff− : *t*(36) = 1.31, *p* = 0.198; cDiff+ : *t*(31) =  − 0.63, *p* = 0.531; cDiff− : *t*(31) =  − 0.75, *p* = 0.456; Gen+ : *t*(36) = 0.37, *p* = 0.716; Gen-: *t*(35) =  − 1.17, *p* = 0.249, indicating that the decrease in standard deviations after associative learning are most likely due to changes in sensitivity for the differences in orientations.

### CS-rating task

**Figure 5:**
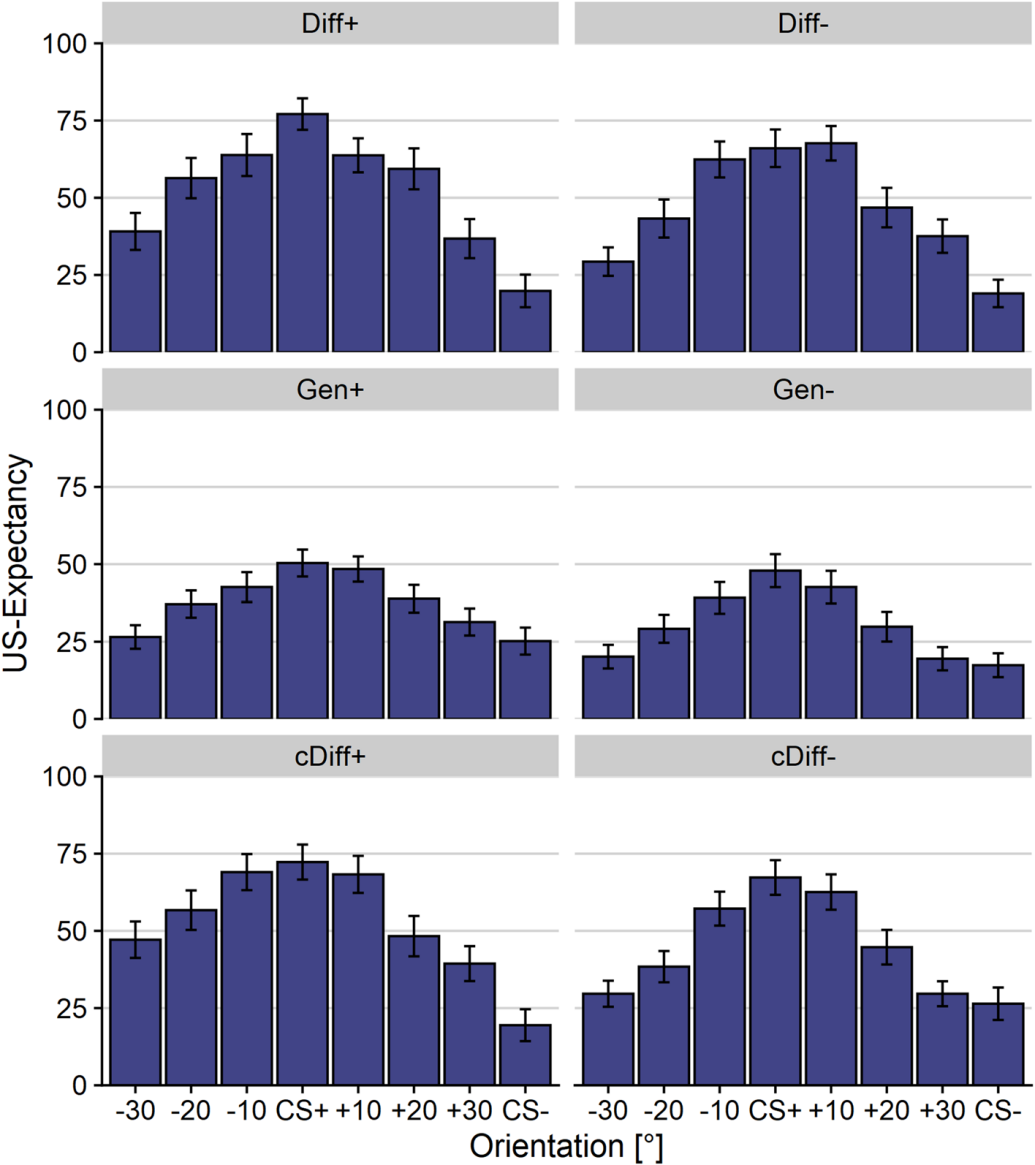
Mean US-expectancy ratings to the conditioned and generalization stimuli after associative learning. All groups demonstrated the expected response pattern for US-expectancy ratings. After associative learning, generalization learning groups showed generally lower US-expectancy ratings than differential learning groups. Error bars depict the standard error of the mean.

*US-expectancy ratings:* Regarding US-expectancy, the 2 (learning type) × 2 (aversiveness) × 8 (orientation) ANOVA revealed a marginal significant main effect of aversiveness, *F*(1, 128) = 3.70, *p* = 0.056, *η*_*p*_^2^ = 0.03 [CI 0.00; 0.10], indicating slightly higher US-expectancy ratings for groups with aversive compared to neutral US (see Fig. 5). In addition, the main effect of learning type, *F*(1, 128) = 25.87, *p* < 0.001, *η*_*p*_^2^ = 0.17 [CI 0.07; 0.28], orientation, *F*(5.61, 718.43) = 37.90, *p* < 0.001, *η*_*p*_^2^ = 0.23 [CI 0.18; 0.27], and the interaction between orientation and learning type, *F*(5.61, 718.43) = 3.69, *p* = 0.002, *η*_*p*_^2^ = 0.03 [CI 0.01; 0.05], were significant*.* All groups showed the expected generalization gradient around the CS+. However, while differential learning groups demonstrated higher US-expectancy ratings for all orientations compared to the CS− orientation [CS+ : *t*(64) = 8.89, *p* < 0.001, *d* = 1.10; GS+ 10°: *t*(64) = 8.29, *p* < 0.001, *d* = 1.03; GS-10°: *t*(64) = 7.32, *p* < 0.001, *d* = 0.91; GS+ 20°: *t*(64) = 5.25, *p* < 0.001, *d* = 0.65; GS-20°: *t*(64) = 4.84, *p* < 0.001, *d* = 0.60; GS+ 30°: *t*(64) = 3.03, *p* = 0.004, *d* = 0.38; GS-30°: *t*(64) = 2.70, *p* = 0.009, *d* = 0.33], generalization learning groups only revealed higher ratings for all orientations compared to the CS− except for the + 30° and − 30° orientations [CS+ : *t*(66) = 5.08, *p* < 0.001, *d* = 0.62; GS+ 10°: *t*(66) = 4.74, *p* < 0.001, *d* = 0.58; GS-10°: *t*(66) = 4.32, *p* < 0.001, *d* = 0.53; GS+ 20°: *t*(66) = 2.79, *p* = 0.007, *d* = 0.34; GS-20°: *t*(66) = 2.56, *p* = 0.013, *d* = 0.31; GS+ 30°: *t*(66) = 1.32, *p* = 0.191, *d* = 0.16; GS-30°: *t*(66) = 0.55, *p* = 0.583, *d* = 0.07]. The 2 (target) × 2 (aversiveness) × 8 (orientation) ANOVA mainly confirmed these results, revealing significant main effects of aversiveness, *F*(1, 125) = 3.94, *p* = 0.049, *η*_*p*_^2^ = 0.03 [CI 0.00; 0.11], and orientation, *F*(4.98, 621.90) = 48.58, *p* < 0.001, *η*_*p*_^2^ = 0.28 [CI 0.23; 0.32]. No effect involving the factor target was significant, *p*s > 0.586.

*Threat ratings:* Corresponding results for threat ratings can be found in the supplemental material.

## Discussion

The aims of this study were to establish a web-based aversive learning and discrimination task, which yields a continuous measure of discrimination acuity, and to test the hypothesis that aversive learning changes visual discrimination. To this end, we measured visual discrimination before and after differential associative learning, using grating stimuli varying in orientation as conditioned stimuli. In addition, there were three types of control group conditions. Participants in the first control conditions were presented with additional six grating stimuli during associative learning, similar to the study by McTeague et al.^10^. Participants in the second control conditions were psychophysically tested on the CS− instead of the CS+. And thirdly, for each of these groups, half of the participants were presented with neutral US, while the other half saw aversive US.

As expected, the US-validation task demonstrated successful picture stimulus manipulation. Groups that were confronted with aversive pictures rated the US as more arousing and more unpleasant than groups with neutral US, consistent with normative ratings of these pictures^28^. Crucially, groups were able to memorize the US equally well, suggesting that participants paid adequate attention throughout the associative learning paradigm. Furthermore, threat and US-expectancy ratings revealed the expected generalization gradients after associative learning^10,39,40^. All groups expected the US more frequently to appear after CS+ compared to CS− presentations, while expectancies decreased with increasing dissimilarity from the CS+. Correspondingly, perceived threat was increased for participants in the groups seeing aversive US only (see supplemental material).

It has long been assumed that learning to predict threat prompts enhanced perceptual discrimination for threat-signaling stimuli^9^. However, recent studies show mixed results with both enhanced^23^ and impaired^22^ discrimination acuity after aversive learning. In our study, participants in the differential learning groups demonstrated narrower response distributions after associative learning, suggesting that associative learning sharpens visual discrimination, and thus, improves discrimination acuity. Contrary to expectations, this effect was not specific for aversive US, but could also be found in the differential learning group with neutral US. These results suggest that general stimulus salience, rather than threat-specific processes, prompt enhanced visual discrimination acuity. Pairing the CS with a complex, although neutral, picture stimulus, increases its salience since it is predicting a novel event^41,42^. This is especially true for between-group comparisons, where participants either see the aversive or the neutral stimuli. However, we cannot exclude that participants in the neutral condition did not expect also aversive US in upcoming trials, as they were not told about viewing one category of US only. Consequently, the CS in the neutral condition might have gained motivational significance and thus salience. Crucially, motivationally significant stimuli receive prioritized attentional processing^43,44^. Our data add to a vast body of results from the visual cognition literature, showing evidence that learned predictiveness is associated with facilitated target recognition during an attentional blink task^45^, decreased response times in dot probe tasks^46,47^, and increased processing speed during a visual recognition paradigm independent of stimulus valence^48^. In summary, these and our results demonstrate converging evidence that learned predictiveness of a stimulus produces an attentional bias towards these stimuli^49^.

It is also important to mention that the narrowing of the response distributions in the visual discrimination task most likely reflects increased sensitivities to the angular distance between the test and target orientations^10,23,25^. In contrast, a general response bias would result in generally less ‘identical’-responses to all orientations, including target orientations. However, such a response bias would lead to a consistent vertical offset in the response distribution without altering its width (standard deviation). In addition, explicit comparisons of the absolute number of ‘identical’-responses to the target stimuli revealed no changes due to associative learning, further supporting the idea of increased sensitivities to the differences from US-associated orientations. This is also in line with the notion that associative learning leads to improved discrimination among motivationally significant stimuli rather than generally reduced decision thresholds, risking—potentially fatal—misses^20,50^.

On a neural level, it is well established that learning to detect threat-related signals is associated with enhanced activity in visuocortical areas^9^. In particular, it has been demonstrated that aversive learning induces neural orientation tuning in the primary visual cortex, leading to sparser neural representations of conditioned threat^10,25^. Yet, the mechanisms driving these changes are still under debate. It has been assumed that re-entrant projections from fear-relevant areas centered around the amygdala prompt short- and long-term adaptions in threat-related feature-specific neurons^9,51^. However, a recent study, exploiting the benefits of simultaneous fMRI-EEG analysis, showed that threat-related visuocortical changes were associated with neural activity in the ventral attention network, but not in the amygdala^52^. The ventral attention network, including the temporoparietal and inferior frontal cortices, is specialized on detecting behaviorally relevant, salient stimuli^53^. The results of our current study are well in line with the notion that the ventral attention network, activated by salient stimuli via associative learning, induces visuocortical tuning in orientation-specific neurons, which then leads to improved discrimination acuity in behavioral measures. Crucially, these changes do not depend on fear-relevant networks, which might be less activated by the neutral US used in the present study.

To support the hypothesis that stimulus predictiveness through associative learning leads to improved visual discrimination, we compared the experimental groups with control conditions, which were psychophysically tested on the unpaired stimulus (CS−) instead of the CS+ . Crucially, the control conditions did not exhibit improved visual discrimination. Thus, enhanced discrimination acuity in the differential learning groups can neither be explained by training effects nor by sensitization, since the control groups were presented with the same US and the same amount of CS as the experimental group. These results are also in line with the findings of Rhodes et al.^23^, demonstrating reduced discrimination thresholds for the CS+, but not for stimuli that were shifted by 90° relative to the CS+.

The present study also included two further groups that did the same discrimination task as the differential learning groups but were presented with six additional generalization stimuli (GS) in 10° steps around the CS+^10^ during the associative learning paradigm. These participants experienced directly that only the CS+ was followed by an US, while all other GS, even those very similar to the CS+ , signaled safety. Consequently, we assumed that the generalization learning groups would evidence even higher discrimination acuities after learning since they were able to make a more discriminative learning experience compared to the differential learning groups. Yet, participants in the generalization learning groups did not exhibit changes in visual discrimination. On the contrary, analysis of US-expectancy even demonstrated generally reduced and a less distinct pattern of expectancy ratings to the CS and GS compared to the differential learning groups, indicating that including additional GS to the learning paradigm led to reduced, instead of improved discrimination. One simple explanation is that the generalization learning groups saw each stimulus only ten times compared to 40 CS presentations in the differential learning groups. Here, our goal was to keep the total number of stimulus presentations equal in all groups. As a result, however, there were only ten US presentations as well, which could have led to reduced associative strength compared to differential learning groups, who saw 40 US^54^. In addition, there is evidence that changes in perceptual processing of threat occurs sometimes after extensive aversive learning only^55^. On the other hand, it could be shown that visuocortical activity to conditioned threat increased after as few as two CS presentations^10^. It is important to mention, however, that the authors used a fully instructed aversive learning paradigm and that the extent to which these neural changes translate to behavioral measures of discrimination acuity still warrants further research.

It is important to note that this study was conducted completely remotely, limiting controllability over external factors as the hard- and software used by the participant (e.g., display size), as well as environment variables (e.g., distractors), which are usually standardized in laboratory studies. However, this can also be considered as a strength of the current study since we found significant changes in visual discrimination despite these limitations. Please note, that the design of the current study does not allow a disentangling of how strongly the effects on visual discrimination are driven by stimulus salience compared to affective properties of the stimulus. To further investigate these competing hypothesis, future studies could either associate both CS+ and CS− with US of different valence, thereby controlling for stimulus predictiveness, or they could experimentally manipulate the strength of stimulus predictiveness by varying the reinforcement rates of conditioned stimuli (e.g., 0% vs. 50% vs. 100% CS-US associations). We also had a considerable number of dropouts during the web-based paradigm. Crucially, as no data from incomplete datasets were saved, we cannot determine at which stage of the paradigm those dropouts occurred. It seems reasonable to assume that most participants aborted the experiment during the instruction of the main tasks, however, we cannot exclude that at least some participants dropped out due to the aversiveness of the unpleasant US, resulting in a potential selection bias, which is generally less controllable in web-based compared to laboratory studies. In addition, the US-validation and memory tasks confirmed that participants paid adequate attention to the US and perceived them according to their normative ratings. This is well in line with studies demonstrating that cognitive and affective processes can be validly studied with minimal costs using smartphone- or web-based paradigms^26,27,56,57^.

In conclusion, the present web-based study presents evidence that associative learning improves visual discrimination acuity. These changes are likely to be driven by enhanced stimulus salience through acquired predictiveness. However, future studies need to replicate these psychophysical findings and seek converging evidence from direct neurophysiological measures of electrocortical and functional imaging studies.

## Supplementary Information

Supplementary Information.

## Data Availability

All data and analyses are publicly available at https://osf.io/4cz2e.
