# Supplementary material for: Associative learning shapes visual discrimination in a web-based classical conditioning task
Source: Sci Rep. 2021 Aug 3;11:15762. doi: 10.1038/s41598-021-95200-6 (PMC8333260; doi:10.1038/s41598-021-95200-6)
Supplement: Supplementary file 1 — Supplementary Information. [file 41598_2021_95200_MOESM1_ESM.docx]

**Supplemental material for:**

**Associative learning shapes visual discrimination in a web-based classical conditioning task**

Yannik Stegmann^1^, Marta Andreatta^1,2^, Paul Pauli^1,3^, Matthias J. Wieser^2^

^1^ Department of Psychology (Biological Psychology, Clinical Psychology, and Psychotherapy), University of Würzburg, Germany

^2^ Department of Psychology, Education, and Child Studies, Erasmus University Rotterdam, Netherlands

^3^ Center for Mental Health, Medical Faculty, University of Würzburg, Germany

**Results**

**Discrimination task – standard deviations extracted from norm distributions**

To test the robustness of our analyses, we re-run all tests with standard deviations extracted from norm distributions instead of Cauchy distributions.

The 2 (learning type: differential vs generalization learning) x 2 (aversiveness: neutral vs aversive US) x 2 (phase: pre vs post) ANOVA yielded a main effect of phase, *F*(1, 137) = 7.40, *p* = .007, *η_p_^2^* = .05 [CI: 0.00; 0.14], and a marginal main effect of learning type, *F*(1, 137) = 3.27, *p* = .073, *η_p_^2^* = .02 [CI: 0.00; 0.09], which were further qualified by an interaction effect between phase and learning type, *F*(1, 137) = 4.81, *p* = 0.030, *η_p_^2^* = .03 [CI: 0.00; 0.11]. Other effects were not significant, *p*s > .365. Post-hoc *t*-tests revealed a decrease in standard deviations, and therefore, improved discrimination acuity after associative learning for the groups Diff+, *t*(30) = -2.55, *p* = .016, *d* = -0.46 [CI: -0.83; -0.08], and Diff-, *t*(36) = -3.10, *p* = .004, *d* = -0.51 [CI: -0.85; -0.16], but not for Gen+, *t*(36) = 0.08, *p* = .936, *d* = 0.01 [CI: -0.31; 0.34], and Gen-, *t*(35) = -0.61, *p* = .545, *d* = -0.10 [CI: -0.43; 0.23]. These results indicate sharpened visual discrimination after associative learning in the differential learning groups, but not in the generalization learning groups, independent of the US valence.

The 2 (target: CS+ vs CS-) x 2 (aversiveness: neutral vs aversive US) x 2 (phase: pre vs post) ANOVA revealed a marginal effect of phase, *F*(1, 128) = 3.07, *p* = .082, *η_p_^2^* = .02 [CI: 0.00; 0.10], which was again further qualified by a significant interaction between target and phase, *F*(1, 128) = 8.74, *p* = .004, *η_p_^2^* = .06 [CI: 0.01; 0.16]. No other effect was significant, *p*s > .368. The control groups showed no changes in discrimination acuity due to associative learning, cDiff+: *t*(31) = -0.18, *p* = .861, *d* = -0.03 [CI: -0.38; 0.32], cDiff-: *t*(31) = 1.22, *p* = .231, *d* = 0.22 [CI: -0.14; 0.56].

**Threat ratings:**

After associative learning, the 2 (aversiveness) x 2 (learning type) x 8 (orientation) ANOVA yielded a significant two-way interaction between aversiveness and orientation, *F*(5.04, 600.15) = 12.64, *p* < .001, *η_p_^2^*= .10 [CI: 0.06; 0.13], while all effects involving learning type were not significant (*p*s > .237). Before associative learning, no differences among groups and conditions could be found (*p*s > .120). Similar results could be retrieved for the 2 (aversiveness) x 2 (target) x 8 (orientation) ANOVA after learning, revealing a significant interaction between aversiveness and orientation, *F*(4.88, 575.28) = 17.90, *p* < .001, *η_p_^2^*= .13 [CI: 0.09; 0.17], without any effect involving the factor target (*p*s > .276). These results suggest that the groups that saw aversive US exhibited the expected generalization gradient around the CS+, while groups that saw neutral US did not (see Fig. 1S).


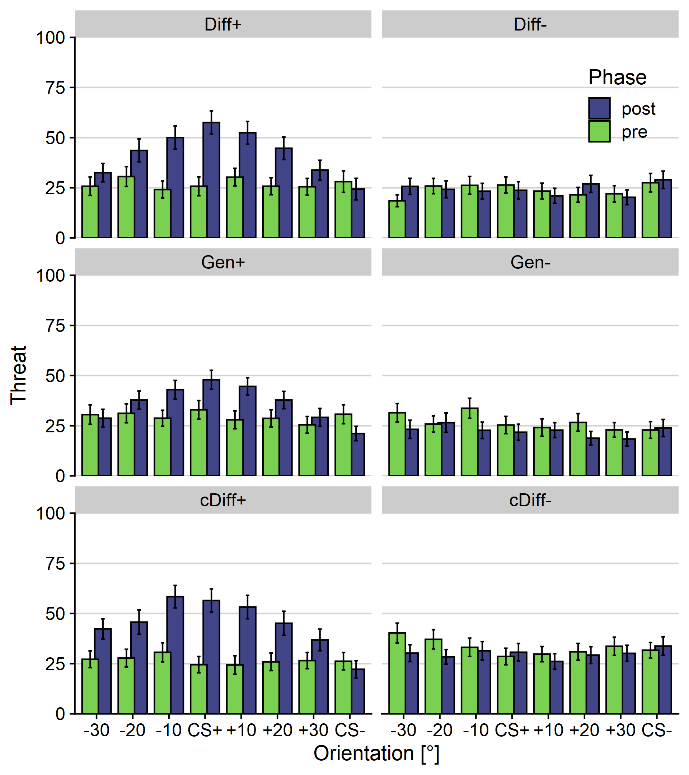


Figure 1S: Mean subjective threat ratings to the conditioned and generalization stimuli. Only groups with aversive US demonstrated the typical generalization gradient around the CS+ after associative learning. Error bars depict the standard error of the mean.
